# Coding-Complete Genome Sequence of a Recombinant Human Norovirus Strain Identified as Subtype GII.p12_GII.3

**DOI:** 10.1128/MRA.01385-19

**Published:** 2020-01-30

**Authors:** Byung-Joo Park, Hee-Seop Ahn, Sang-Hoon Han, Hyeon-Jeong Go, Eu-Lim Lyoo, Changsun Choi, Jinjong Myoung, Joong-Bok Lee, Seung-Yong Park, Chang-Seon Song, Sang-Won Lee, In-Soo Choi

**Affiliations:** a Department of Infectious Diseases, College of Veterinary Medicine, Konkuk University, Seoul, Republic of Korea; b Department of Food and Nutrition, College of Biotechnology and Natural Resources, Chung-Ang University, Anseong, Republic of Korea; c Korea Zoonosis Research Institute, Chonbuk National University, Jeonju, Republic of Korea; DOE Joint Genome Institute

## Abstract

A human norovirus (HuNoV) strain was obtained from a patient with acute gastroenteritis, and its complete coding sequence was determined. The coding-complete viral genome, with three open reading frames, was 7,565 bp long, with a GC content of 49.9%. The genotype of the HuNoV strain obtained in this study was identified as GII.p12_GII.3.

## ANNOUNCEMENT

Norovirus, a viral pathogen that causes gastroenteritis in humans, results in 19 to 21 million total illnesses and 56,000 to 71,000 hospitalizations every year in the United States (1). Human norovirus (HuNoV) belongs to the family *Caliciviridae* and contains a 7.3- to 7.7-kb single-strand positive-sense RNA genome (2). Noroviruses include seven genogroups (GI to GVII) classified based on the nucleotide sequence of the viral capsid gene (VP1), and GI, GII, and GIV noroviruses can infect humans (3, 4). Each genogroup is further divided into genotypes based on the phylogenetic clustering of VP1 amino acid sequences (5). Approximately 70% of HuNoV infections in 2010 were caused by GII.4 noroviruses, and studies in 2018 reported that infections with GII.17 noroviruses (30.2%) were second only to infections with GII.4 noroviruses (47.3%) (6, 7). Recombinant GII.p12 subtypes containing an RNA-dependent RNA polymerase (RdRp) gene derived from GII.12 and an open reading frame 2 (ORF2) encoding a major capsid protein originating mainly from GII.3, GII.4, or GII.13 have been reported in several countries (8–10). The Sanger sequencing method can be used to determine the partial viral genomic sequences only when the partial nucleotide sequences are known; therefore, high-throughput sequencing has been used to analyze the whole genomes of unknown or partially known pathogens (11–13).

We obtained HuNoV strain CAU140599 in 2014 from a Korean patient with acute gastroenteritis. The viral particles were purified from the supernatant of the patient’s fecal sample using discontinuous sucrose gradient (10 to 60%) ultracentrifugation at 100,000 × *g* for 16 h, as described previously (14). Extraction of viral RNA and construction of a library were completed with the TruSeq RNA sample preparation kit version 2 (Illumina, Inc., CA, USA). High-throughput sequencing was performed using the HiSeq X Ten reagent kit version 2.5 (151-bp reads; Illumina). Reads were trimmed using Trimmomatic version 0.38 (mean quality score, 15; sliding window size, 4) (http://www.usadellab.org/cms/?page=trimmomatic). Trimmed reads (42,524,862 reads) were obtained and assembled using Trinity version trinityrnaseq_r20140717 with the default parameters (https://github.com/trinityrnaseq/trinityrnaseq/wiki). The assembled contigs were filtered and clustered into nonredundant transcripts using CD-HIT-EST version 4.6 (http://weizhongli-lab.org/cd-hit). Viral annotation and prediction of ORFs were performed by searching for the contig sequences in the KO_Virus_Nucl database (version 20190104) (http://www.genome.jp/kegg/ko.html) using BLASTX version 2.4.0+ (https://blast.ncbi.nlm.nih.gov/Blast.cgi) and DIAMOND version 0.9.21 (https://github.com/bbuchfink/diamond), with a cutoff E value of 1.0E−5. The coding-complete genome of NoV, containing three ORFs, had 19,641.2× coverage with 1,001,204 reads.

**FIG 1:**
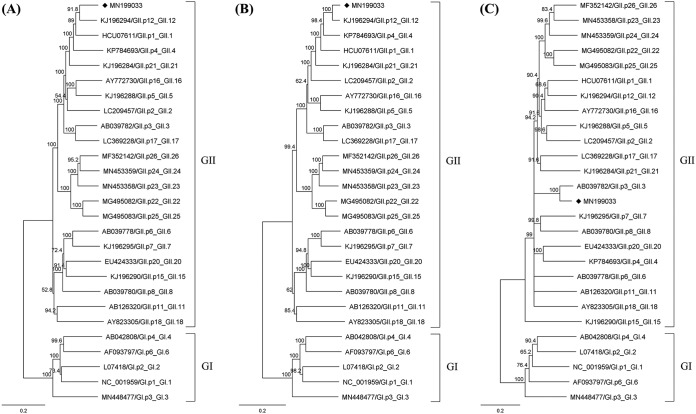
Phylogenetic analysis of HuNoV using the complete genome, ORF1, and ORF2. The coding-complete genome (A), ORF1 (B), and ORF2 (C) nucleotide sequences of the CAU140599 strain were analyzed along with those of other HuNoV strains. Phylogenetic trees were constructed using the neighbor-joining method with the Tamura-Nei genetic distance model in Geneious version 10.2.6. The nucleotide sequences were aligned by multiple alignment using fast Fourier transform (MAFFT) with the default parameters in Geneious. Bootstrap analysis was performed with 500 cycles, and bootstrap values greater than 50% are presented at the branch points. Norovirus genotypes were annotated by Norovirus Typing Tool version 2.0 (https://www.rivm.nl/mpf/typingtool/norovirus).

The coding-complete genome sequence of the CAU140599 strain contained 7,565 bp, with a GC content of 49.9%. ORF1, coding for nonstructural proteins such as RdRp, ORF2, coding for a major structural protein (VP1), and ORF3, coding for a minor structural protein (VP2), were positioned at bp 2 to 5101, bp 5082 to 6728, and bp 6728 to 7492, respectively. When the coding-complete sequence of the CAU140599 strain was compared to the sequences deposited in GenBank by phylogenetic analysis using Geneious version 10.2.6, it presented 94.40% nucleotide identity to strain GII/Hu/JP/2000/GII.P12_GII.12/Saitama/KU16 (GenBank accession no. KJ196294) (Fig. 1A).

Further phylogenetic analysis using the ORF1 sequence of the CAU140599 strain indicated that it belonged to the GII.p12_GII.12 genotype (Fig. 1B). ORF1 of the CAU140599 strain displayed 84.75% nucleotide identity to that of a GII.p12_GII.12 strain (Fig. 1B). Interestingly, ORF2 of the CAU140599 strain was classified in the GII.3 genotype based on phylogenetic analysis (Fig. 1C). The ORF2 sequence of the CAU140599 strain was 88.66% identical to that of the GII.3 genotype. In recombinant analysis using the recombination detection program RDP version 4.97 (http://web.cbio.uct.ac.za/~darren/rdp.html), recombination between the ORF1 region (bp 1 to 5082) of a GII.p12_GII.12 strain (GenBank accession no. KJ196294) and the ORF2 region (bp 5083 to 7534) of a GII.p3_GII.3 strain (GenBank accession no. AB039782) was identified. Therefore, the genotype of the HuNoV strain obtained in this study was eventually identified as the GII.p12_GII.3 subtype. ORF3 of the HuNoV strain analyzed in this study showed 90.45% identity to the strains of the GII.p3_GII.3 genotype.

### Data availability.

The coding-complete genome sequence of the CAU140599 strain was deposited in GenBank under accession no. MN199033. The raw sequencing reads were submitted to the SRA under accession no. SRX7118213 (BioSample no. SAMN13229199).
